# Fatty acid metabolism is related to the immune microenvironment changes of gastric cancer and RGS2 is a new tumor biomarker

**DOI:** 10.3389/fimmu.2022.1065927

**Published:** 2022-12-14

**Authors:** Shifeng Yang, Boshi Sun, Wenjing Li, Hao Yang, Nana Li, Xinyu Zhang

**Affiliations:** ^1^ Department of General Surgery, The Second Affiliated Hospital of Harbin Medical University, Harbin, China; ^2^ The Key Laboratory of Myocardial Ischemia, Ministry of Education, Harbin, China; ^3^ Department of Otorhinolaryngology, Head and Neck Surgery, Second Affiliated Hospital of Harbin Medical University, Harbin, China

**Keywords:** gastric cancer, fatty acid, immunotherapy, tumor microenvironment, machine learning

## Abstract

**Background:**

Alterations in lipid metabolism promote tumor progression. However, the role of lipid metabolism in the occurrence and development of gastric cancer have not been fully clarified

**Method:**

Here, genes that are related to fatty acid metabolism and differentially-expressed between normal and gastric cancer tissues were identified in the TCGA-STAD cohort. The intersection of identified differentially-expressed genes with Geneset was determined to obtain 78 fatty acid metabolism-related genes. The ConsensusClusterPlus R package was used to perform differentially-expressed genes, which yielded divided two gastric cancer subtypes termed cluster 1 and cluster 2.

**Results:**

Patients in cluster 2 was found to display poorer prognosis than patients in cluster 1. Using machine learning method to select 8 differentially expressed genes among subtypes to construct fatty acid prognostic risk score model (FARS), which was found to display good prognostic efficacy. We also identified that certain anticancer drugs, such as bortezomib, elesclomol, GW843682X, and nilotinib, showed significant sensitivity in the high FARS score group. RGS2 was selected as the core gene upon an analysis of the gastric cancer single-cell, and Western blotting and immunofluorescence staining results revealed high level of expression of this gene in gastric cancer cells. The results of immunohistochemical staining showed that a large amount of RGS2 was deposited in the stroma in gastric cancer. A pan-cancer analysis also revealed a significant association of RGS2 with TMB, TIDE, and CD8+ T-cell infiltration in other cancer types as well. RGS2 may thus be studied further as a new target for immunotherapy in future studies on gastric cancer.

**Conclusion:**

In summary, the FARS model developed here enhances our understanding of lipid metabolism in the TME in gastric cancer, and provides a theoretical basis for predicting tumor prognosis and clinical treatment.

## 1 Introduction

Gastric cancer (GC) is one of the most prevalent malignant digestive system tumors, characterized by a high degree of heterogeneity, difficulty of treatment, and a poor prognosis (1, 2). The liver is the most frequently affected organ by hematogenous metastases of gastric cancer tumors, after liver metastasis, the survival rate was only 20% (3) The development of neoadjuvant chemotherapy and immunotherapy for gastric cancer treatment in recent years has led to improvements in the diagnosis and prognosis of gastric cancer to a certain extent, yet further improvement is still necessary (4). To this end, new tumor markers, therapeutic targets, and treatment strategies need to be developed (5). Previous studies have shown that the occurrence, proliferation, and metastasis of tumors are closely related to their microenvironment. Various tumor cell metabolites can affect the activation of surrounding immune cells in various ways, and suppress their antitumor activity. Alterations in the tumor microenvironment promotes proliferation and development of tumor cells (6). Growing evidence suggests that reprogramming of energy metabolism towards e.g. lactic acid production and acetylation enzymes contributes to the progression of gastric cancer (7). An in-depth investigation of metabolic changes in the tumor microenvironment of gastric cancer may thus provide with a new marker or therapeutic target to improve gastric cancer prognosis and treatment.

In lipid metabolism and especially fatty acid (FA) synthesis, nutrients are converted into metabolic intermediates for membrane biosynthesis, energy storage, and signal molecule production (8). Alterations in lipid metabolism is a hallmark and metabolic phenotype of cancer cells. Blocking the supply of lipids to cancer cells has a significant impact on cancer cell bioenergetics, membrane biosynthesis, and intracellular signal transduction (9). Most tumors were previously shown to display an abnormal lipid metabolism (10). Polymorphonuclear myelogenous suppressor cells (PMN-MDSCs) are pathologically-activated neutrophils that play an important role in the regulation of cancer immune response.The selective pharmacological inhibition of FATP2 was also found to eliminate the activity of PMN-MDSCs, and significantly delay tumor progression in mice. Inhibition of PMN-MDSCs thus improves the efficiency of cancer treatment (11, 12). Therefore, targeted fatty acid-induced oxidative stress can prevent cancer-induced cachexia.

In recent years, inhibition of FA synthesis has attracted attention as a potential strategy for cancer treatment, yet it is not yet implemented in clinical practice (13). The role of lipid metabolism in gastric cancer has also not been widely studied previously. Therefore, we conducted an in-depth study here on the expression and significance of fatty acid disorder-related genes in gastric cancer. We identified differentially-expressed fatty acid metabolism-related genes in gastric cancer, and determined two subtypes based on consistency clustering analysis. A prognostic signature (FRAS) model was constructed by performing a univariate Cox regression analysis of differentially-expressed genes in different subtypes, and used as a potential molecular marker of gastric cancer to identify immune infiltration and genomic instability patterns. FeaturePlot visualization was performed to display the expression and distribution of model genes in the cell population and to verify the accuracy of the model. A “core gene”, *RGS2* was selected for subsequent experiments, and the relationship between the expression level of RGS2 protein and the prognosis of patients with gastric cancer was evaluated. Finally, we also discussed the biological significance of the *RGS2* gene in multiple cancer types to fully understand the role of fatty acid metabolism in gastric cancer, and to provide a theoretical basis for effective treatment.

## 2 Materials and methods

### 2.1 Patients and tissues samples

All patients were admitted to the Second Affiliated Hospital of Harbin Medical University between May 2020 and June 2022, and diagnosed by pathological examination.Pathological diagnosis was based on the 8th edition of the American Joint Commission on Cancer (14). All participants have informed consent. The study design was approved by the Internal Audit and Ethics Committee of the Second Affiliated Hospital of Harbin Medical University (No : KY2021-075).

### 2.2 Western blotting

The protein content of the cells was extracted, and the expression of *RGS2* protein was analyzed by Western blotting after the cell density of cultures of AGS, HGC27, MKN-45, MKN-1, and the GES-1 cell lines reached 90%.

### 2.3 Data preparation and processing

STAD clinical information and expression data were obtained from the American Cancer Genome Map Database (TCGA, https://cancergenome.nih.gov/) using the TCGA R package biollinks. Tumor samples with both expression and survival information were retained for follow-up analysis, which included 373 cancer and 32 paracancerous samples. Fatty acid-related genes (Geneset) are derived from fatty acid-related factors (fattyacid) in the MsigDB database (HALLMARK, KEGG, REACTOME). A total of 14 pathways and 342 related genes were identified.

### 2.4 Clustering analysis

An intersection between the identified differentially-expressed genes with Geneset yielded 78 differentially-expressed fatty acid-related genes. Using the ConsensusClusterPlus R package, differentially-expressed genes related to fatty acid disorder were clustered based on Euclidean distance. The maximum number of clusters was set to five, and the clustering method to K-means, in order to find a stable and reliable subgroup classification. The results yielded two subtypes, and the differential gene expression between two subtypes was analyzed (screening condition of the difference was: absolute value of log2FC > 1, P< 0.05).

### 2.5 Construction of prognostic risk model

The genes differentially-expressed between the subtypes were analyzed using univariate Cox regression analysis to identify genes related to the prognosis of subtypes. For this purpose, LASSO penalty Cox regression analysis was used *via* the Rglmnet package to construct a prognostic model to minimize the risk of overfitting. Patient scores were calculated according to the expression levels of the pathway genes and their corresponding regression coefficients.


$$\text{Score}=\sum_{i=0}^{n}\beta i*xi$$

βi: weight coefficient of each gene; χi: expression of each gene (FPKM). Patients were divided into high and low score groups based on the median score, and the survminer R package was used for survival analysis of OS based on high and low scores.

### 2.6 Evaluation of immune cell filtration

The CIBERSORT algorithm provided by the IOBR R package was used to calculate the scores of immune cells in 22 types of tumor microenvironments using the default parameters. Based on the gene expression profile in the TCGA-STAD data, the proportion of immune cell infiltration was calculated.

### 2.7 Single-cell dataset analysis

The Seurat R package, which is single-cell transcriptome analysis tool, was used to analyze the single-cell dataset. The analysis workflow mainly included the steps of constructing objects, data standardization, data dimensionality reduction, clustering, and searching for marker genes. Then, the SingleR R packagewas used to annotate the clustering results obtained from Seurat.

### 2.8 Drug sensitivity

Using the pRRophetic R package and the expression data of model genes, the sensitivity (IC50 value) of 138 drugs in the GDSC database was predicted, and the sensitivity of STAD patients to drug therapy was evaluated based on the predicted IC50 values.

### 2.9 Statistical analysis

The R program (version 4.1.2) was used for statistical analysis. The survival curve was generated using the Kaplan-Meier method, and the differences between groups were compared using the log-rank test. A Cox regression model was used for univariate and multivariate analyses combined with other clinical features to determine the independent prognostic value of the risk score. The R package timeROC was used for time-dependent ROC curve analysis to evaluate the predictive value of prognostic characteristics. ROC analysis was used to evaluate the sensitivity and specificity of the score in predicting prognosis, and the area under the ROC curve (AUC) was considered to judge prognosis. Statistical significance was set at p< 0.05. The same formula is used to calculate verification scores.

## 3 Results

### 3.1 Differential expression of fatty acid related genes in tumor tissues and their biological functions

**Figure 1:**
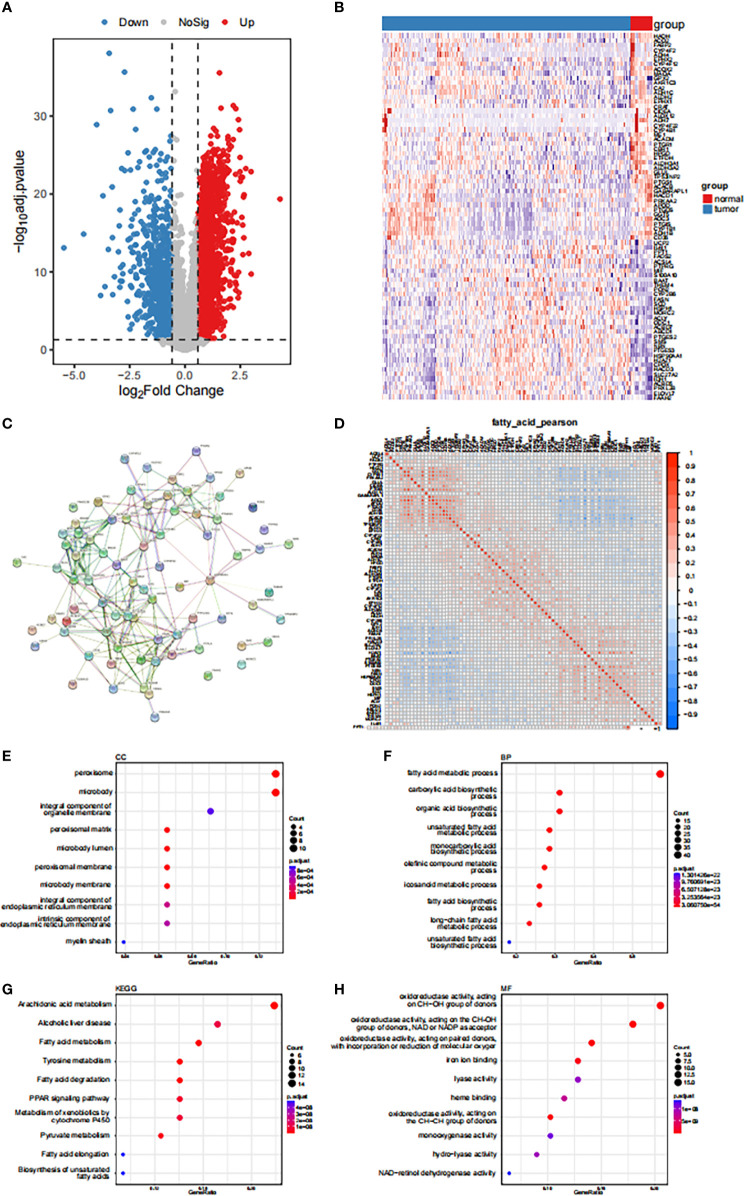
Expression disturbance of fatty acid-related genes in tumors **(A)** Volcanogram of fatty acid related genes differentially expressed in gastric cancer and paracancerous tissues. **(B)** Heat map of differentially expressed fatty acid-related genes. **(C)** Based on the differentially expressed fatty acid related genes, the PPI network was constructed by using STRING database. **(D)** Expression correlation analysis of genes related to differential fatty acids. **(E-H)** GO functional enrichment analysis of genes related to differential fatty acids.

The study population included 373 STAD and 32 paracancerous tissue samples obtained from the TCGA-STAD cohort.|Using log2FC | > 0.585, BH-corrected, and P< 0.05 as differential expression criteria, 3857 genes were found to be differentially-expressed in gastric cancer and paracancerous tissues, with 2801 and 1056 up- and down-regulated genes, respectively. A total of 78 fatty acid-related differentially-expressed genes were identified by determining the intersection of these genes with the Geneset, A volcano map and a differentially-expressed fatty acid metabolism-related gene thermogram is shown in **Figures 1A, B**. The PPI network showed that *HSP90AA1*, *EPHX2*, *ACOX2*, *ACADM*, *ACLY*, and other genes had high connectivity in the network (**Figure 1C**). The correlation between the expression levels of differentially-expressed fatty acid metabolism-related genes was also calculated. Fatty acid metabolism-related genes were found to be classified into three groups (**Figure 1D**). A functional GO enrichment t was found for oxidoreductase activity, acting on the CH-OH group of donors, NAD or NADP as acceptor, acting on paired donors and binding or reducing oxygen molecules, CH-CH group acting on donor, and easy to bind iron ions. These enzymes participate in long-chain fatty acid metabolism, fatty acid biosynthesis, eicosane-like metabolism, olefin metabolism, and unsaturated fatty acid metabolism (**Figures 1E–H**). The clinical feature analysis revealed that there were significant differences in the expression of some fatty acid metabolism-related genes between different age, sex, stage, and grade groups (**Figures S1A-D**).

### 3.2 Determination of molecular subtypes based on fatty acid metabolism related genes

**Figure 2:**
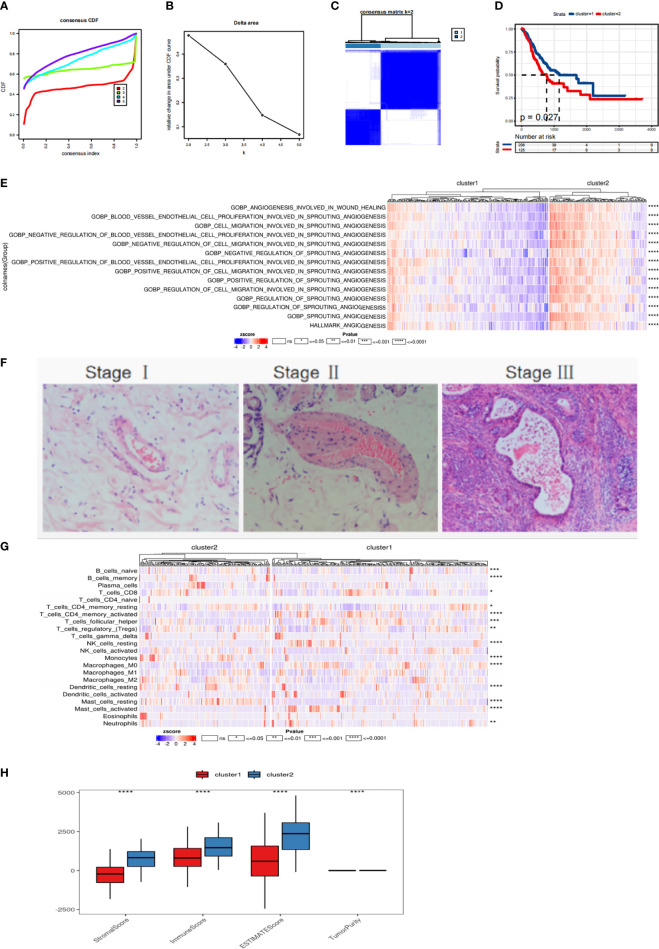
Molecular subtype recognition of fatty acid related genes **(A, B) **TCGA gastric cancer samples were analyzed by consistent cluster analysis based on 78 fatty acid related genes. **(C)** The consistency matrix heat map when the number of clusters is 2. **(D)** Survival curve of patients with fatty acid subtypes. **(E)** The scores of ANGIOGENESIS-related pathways in HALLMARK,GOBP gene set in MSigDB database were calculated by SSGSEA. **(F)** HE staining of gastric cancer tissue. **(G)** Using CIBERSORT to calculate the heat map of immune cell infiltration. **(H)** ESTIMATE was used to calculate immune score, matrix score and tumor purity. ns means p > 0.05, *p<=0.05, **p<=0.01, ***p<=0.001 and ****p<=0.0001.

Subtyping can be used to reveal distinct states of the tumor, and thus help implement personalized treatment strategies. Cancer samples from the TCGA gastric cancer data were subjected to consistency clustering based on expression patterns of 78 different fatty acid metabolism-related genes to identify groups of samples with similar expression patterns. According to the cumulative distribution function and incremental region map of consistent clustering, the change in the CDF curve for the case of two clusters (k = 2, clusters 1 and 2) was found to be close to smooth. Hence, the samples were divided into two subtypes (**Figures 2A-C**). We found that there were significant differences in survival time between patients with different fatty acid metabolism subtypes, and the prognosis of patients in cluster 2 was worse than that of cluster 1 patients (**Figure 2D**). In addition, the scores of angiogenesis-related pathways in the HALLMARK and GOBP gene sets in the MSigDB database were calculated using SSGSEA. The results showed significant differences between the scores of all pathways related to angiogenesis between the fatty acid metabolism related molecular subtypes (**Figure 2E**). A large number of blood vessels (**Figure 2F**) were found in gastric cancer tissues by HE staining. Immune cell infiltration was calculated using CIBERSORT, and the immune score, matrix score, and tumor purity (**Figure 2G**) were calculated using ESTIMATE algorithms. The heat map of immune cell infiltration in subtypes showed that there were significant differences in Mast_cells_activated, Dendritic_cells_resting, Macrophages_M0, etc. Inter-subtype immune scores and matrix scores (**Figure 2H**).

### 3.3 Construction of a fatty acid metabolism-related prognostic signature

**Figure 3:**
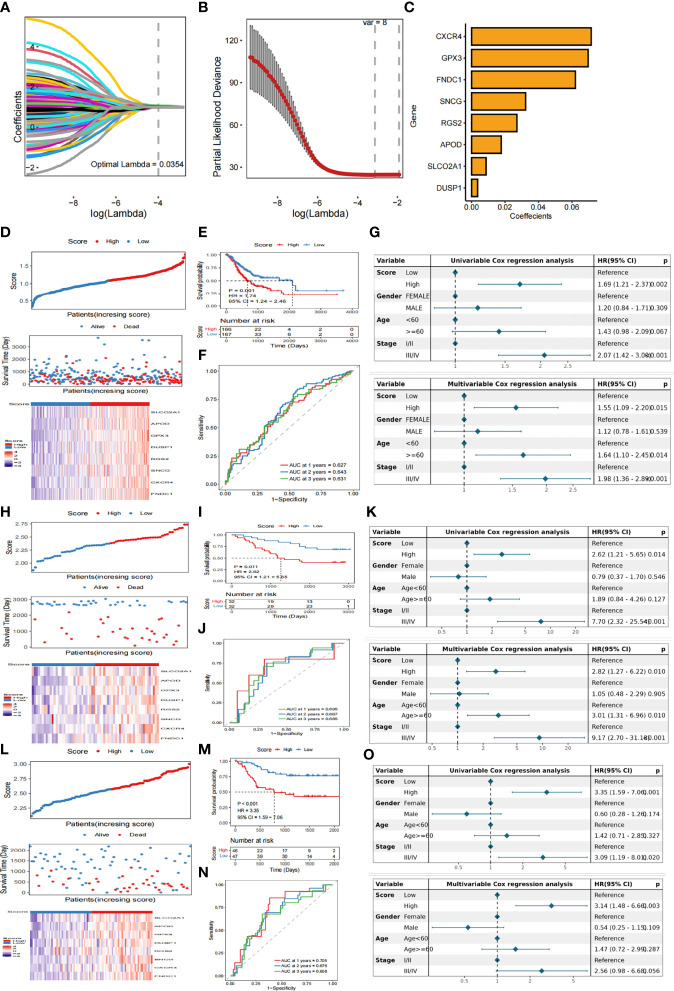
Construction of hierarchical scoring system for Fatty Acid related prognosis **(A)** LASSO coefficient profiles of eight genes. **(B)** Tuning parameter lambda (λ) selected by cross-validation error curve. **(C)** Eight genes determined based on the optimal value of λ **(D)** The relationship between survival status/risk score of TCGA cohort, mRNA expression heat map of 8 genes and survival time (days)/risk score. **(E)** Kaplan-Meier OS analysis of gastric cancer patients in low-risk and high-risk groups **(F)** The time-dependent ROC curve of TCGA training data set OS. AUC was evaluated at 1 year, 3 years and 5 years, respectively. p value was calculated using the log-rank test. p< 0.001. **(G)** Univariate and multivariate Cox analysis were used to determine whether Score was an independent prognostic factor for OS. **(H)** GSE13861 dataset was used to analyze the relationship between survival status/risk score, mRNA expression heat map of 8 genes and survival time (days)/risk score. **(I)** Kaplan-Meier OS analysis of gastric cancer patients in low-risk and high-risk groups based on GSE13861 data set **(J)** Time-dependent ROC curve of GSE13861 dataset OS. AUC was evaluated at 1 year, 3 years and 5 years, respectively. P value was calculated using the log-rank test. P< 0.001. **(K)** The GSE13861 dataset uses univariate and multivariate Cox analysis to determine whether Score is an independent prognostic factor for OS. **(L)** The relationship between survival status/risk score, mRNA expression heat map of 8 genes and survival time (days)/risk score was analyzed by GSE26899 dataset. **(M)** Kaplan-Meier OS analysis of gastric cancer patients in low-risk and high-risk groups based on GSE26899 data set **(N)** Time-dependent ROC curve of GSE26899 dataset OS. AUC was evaluated at 1 year, 3 years and 5 years, respectively. P value was calculated using the log-rank test. P< 0.001. **(O)** The GSE26899 dataset uses univariate and multivariate Cox analysis to determine whether Score is an independent prognostic factor for OS.

We have identified 515 genes differentially-expressed between the two subtypes under the screening condition of | log2FC | > 1 dBH correction p< 0.05. A total of 454 and 61 genes were up- and down-regulated, respectively. Univariate Cox regression analysis showed that 146 genes were associated with OS. KM analysis revealed eight genes (eight genes screened after LASSO-Cox regression analysis) (**Figure S2**). The signature (**Figures 3A-C**) composed of eight genes, and was determined based on the optimal value of λ. The regression coefficient of each gene is shown in **Table S1**.

### 3.4 Verification of prognostic efficacy of FARS based on an analysis of training and external independent verification sets

The score of each patient was calculated according to the formula and the patients were divided into high score group and low score group by the median score. KM curve showed that the survival probability of patients with high score was significantly lower than that of patients with low score (**Figures 3D, E**). To evaluate the predictive efficiency of prognostic models in 1 -, 2 -, and 3-year survival rates, we performed a time-related ROC analysis. The area under the ROC curve (AUC) is 0.627 at 1 year, 0.643 at 2 years and 0.631 at 3 years, indicating that the prediction effect of the model is good (**Figure 3F**). Univariate and multivariate Cox analysis were used to determine whether Score was an independent prognostic factor for OS. In univariate Cox analysis, Score obtained from TCGA data queue was significantly correlated with OS. After correcting other confounding factors, multivariate Cox analysis showed that Score was still an independent predictor of OS (**Figure 3G**).

In order to verify the stability of the model, the Score of each sample is also calculated in GSE13861 dataset and GSE26899 dataset based on the same algorithm. According to the median of Score, gastric cancer samples were divided into high score group and low score group. Consistent with the results obtained by the TCGA cohort, patients with high scores had a lower probability of survival than patients with low scores (**Figures 3H, I, L, M**). In addition ,the prognostic model revealed that the 1- year AUC was 0.695, 2-year AUC was 0.667, 3-year AUC was 0.685 in the GSE13861 dataset (**Figure 3J**),and 1-year AUC was 0.705, 2-year AUC was 0.676, and3-year AUC was 0.658 in the GSE26899 dataset (**Figure 3N**). In the validation set, univariate and multivariate Cox analyses were also used to determine whether Score was an independent prognostic factor for OS. The results show that in univariate Cox analysis, there is a significant correlation between Score and OS. After correcting other confounding factors, multivariate Cox analysis shows that Score is still an independent predictor of OS (**Figures 3K, O**).

### 3.5 FARS is related to the clinical characteristics of tumor

**Figure 4:**
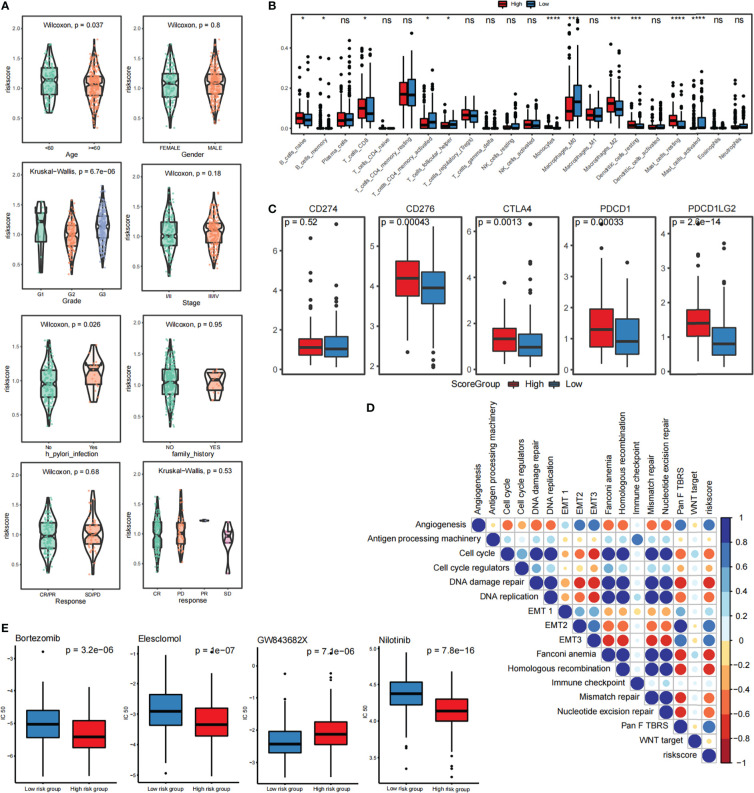
Relationship between risk scoring system and immune infiltration and immunotherapy **(A)** Analysis of correlation between risk score and clinical characteristics of patients with STAD **(B)** CIBERSORT calculated the relationship between immune cell infiltration and risk score **(C)** The difference of gene expression in immune checkpoint between high and low score groups. **(D)** Pearson analysis of SSGSEA score and fatty acid risk score. **(E)** Correlation analysis between fatty acid risk score and chemotherapy resistance. *p<=0.05, ***p<=0.001, ****p<=0.0001, ns means p > 0.05.

We found that the score of patients with *Helicobacter pylori* infection was significantly higher than that of patients without infection and significant differences were also detected between patients with first-, second-, and third-grade cancer: higher grades corresponded to higher scores and poorer prognosis (**Figure 4A**). Immune cell infiltration as calculated by the CIBERSORT algorithm revealed that many immune cell types, such as Mast_cells_activated, Dendritic_cells_resting, and Macrophages_M0, are significantly correlated with the FARS score (**Figure 4B**). **Figure 4C** shows the difference in gene expression of immune checkpoints in the high- and low-risk groups of scores, in which the expression leves of *CD276*, *CTLA4*, *PDCD1*, and *PDCD1LG2* were significantly higher in the high score group. This high expression level helps gastric cancer cells escape immune surveillance and promote immune escape. Based on the calculation of the Pearson correlation between the fatty acid risk score (Score) and the identified gene signature score, we detected several gene sets related to immunity and EMT from the literature, and then performed mapping between the SSGSEA score and the fatty acid risk score (Score) of these samples. We found a significant correlation between the FRAS score and EMT2, EMT3, and PanFTBRS, which promote the EMT process in gastric cancer cells (**Figure 4D**). We further evaluated the relationship between fatty acid risk score and chemotherapeutic drug resistance, and also calculated the difference in chemotherapeutic drug resistance between highFARS and lowFARS using the pRRophetic package. The IC50 values of bortezomib, elesclomol, and nilotinib were found to be significantly different between highFARS and lowFARS, and with stronger chemotherapeutic effects (**Figure 4E**) in the low-score group.

### 3.6 Single-cell dataset analysis

**Figure 5:**
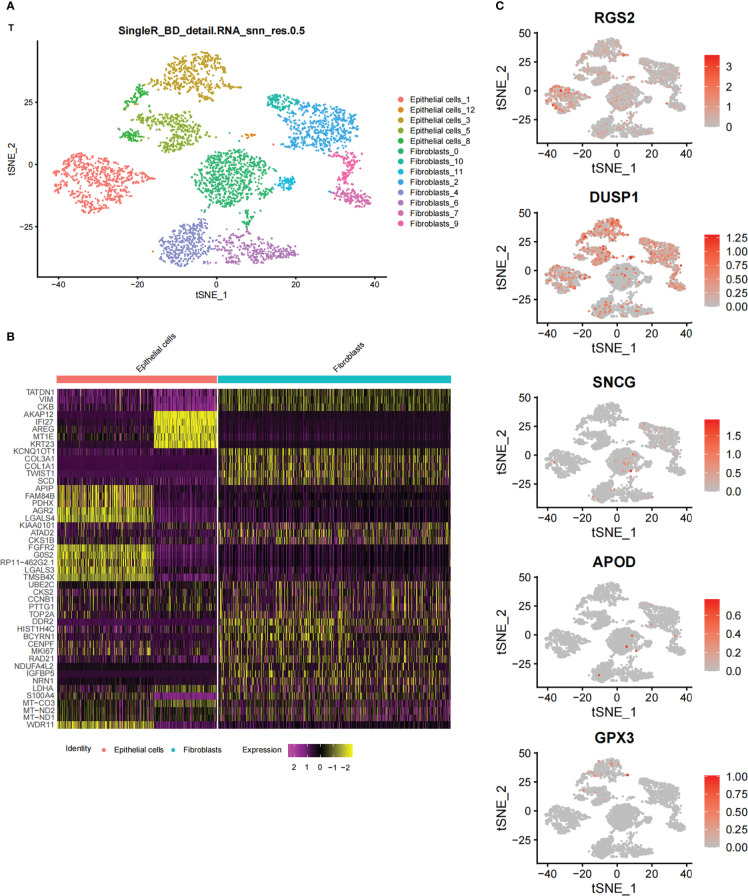
Single cell data set analysis **(A)** T-SNE clustering and cell group annotation based on STAD samples from single-cell data sets. **(B)** Draw a heat map of the Marker gene of TOP5 in each Cluster. **(C)** Display the expression and distribution of model genes in the cell population by FeaturePlot visualization.

Using the STAD samples in the single-cell data set downloaded from the GEO database (GSE142750), the cells were grouped and annotated based on an t-SNE analysis. A total of 107597 cells (33694 features) were grouped into 13 clusters, and finally annotated as two large cell groups (**Figure 5A**). Then, the union of the top 5 marker genes of in each cluster was used to draw a heat map to show the differential expression of each marker gene in each subtype. No genes included in the constructed model (model genes) was detected (**Figure 5B**) among these top 5 marker genes.

Feature plot visualization was used to show the expression and distribution of model genes in the cell population. The results showed that *RGS2* and *DUSP1* were significantly expressed in the cell population, and three model genes, *CXCR4*, *SLCO2A1*, and *FNDC1*, were not in the cluster, indicating that the two model genes that were significantly expressed could be used as marker genes (**Figure 5C**) of cancer.

### 3.7 Biological significance of *RGS2* in gastric cancer

**Figure 6:**
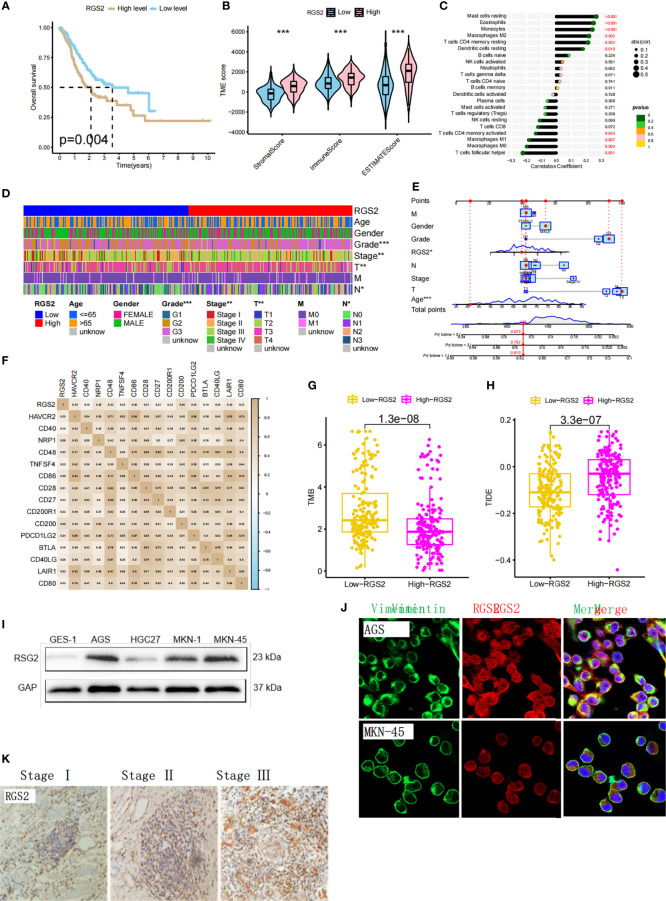
Biological significance of RGS2 in gastric cancer and expression level of gastric cancer cell line **(A)** The relationship between the high and low expression of RGS2 in gastric cancer and prognosis **(B)** The relationship between the high and low expression of RGS2 and the score of TME **(C)** The correlation between the high and low expression of RGS2 and immune cell infiltration. **(D, E)** Analysis of the correlation between the high and low expression of RGS2 and the clinicopathological features of patients **(F)** The relationship between the high and low expression of RGS2 and immune checkpoints **(G)** The relationship between the high and low expression of RGS2 and TMB score **(H)** The relationship between high and low expression of RGS2 and TIDE score **(I)** Expression at mRNA level of RGS2 in gastric cancer cell lines AGS, HGC27, MKN-1, MKN45 and normal gastric mucosal cells GES-1 **(J)** Expression and localization of RGS2 in gastric cancer cell lines AGS and MKN45 **(K)** Expression of RGS2 in different clinical stages of gastric cancer. ***p<=0.001.

We found that the high expression of *RGS2* in gastric cancer was significantly correlated with a shorter survival time (**Figure 6A**). The TME score showed that the high expression of *RGS2* was positively correlated with the stomalscore, Immunescore, and Estimatescore, which indicated a worse immune response (**Figure 6B**). Correlation analysis of immune cell infiltration showed that the expression of 10 types of immune cells in 22 types of immune cells was correlated with *RGS2* expression (**Figure 6C**). We also analyzed the clinicopathological features of patients with high and low *RGS2* expression, including age, sex, survival, grade grade, T stage, and N stage. The figure shows that there is no statistical difference in age and sex between the high and low *RGS2* expression groups. High *RGS2* expression was found to be closely related to poor prognosis. This finding shows that high expression of *RGS2* represents a higher degree of malignancy based on clinicopathological features (**Figures 6D, E**). We also analyzed the relationship between expression levels of *RGS2* and immune checkpoints (**Figure 6F**). We found that the lower tumor mutation load in the group with high expression of *RGS2* increased the difficulty of receiving the benefit of immune checkpoint inhibitors for patients (**Figure 6G**). We found that the TIDE score of the *RGS2* high expression group was significantly higher than that of low expression group (**Figure 6H**). This also indicates that high *RGS2* expression is more likely to lead to immune dysfunction and immune rejection. We have determined the mRNA and protein expression levels of *RGS2* in GES-1 gastric mucosal cells and AGS, HGC-27, MKN-1, and MKN-45 gastric cancer cell lines. Accordingly, the expression of RGS2 in gastric cancer cell line was found to be higher than that of GES-1 (**Figure 6I**) at both mRNA level and protein level. Immunofluorescence staining showed that RGS2 was highly expressed in gastric cancer cell lines AGS and MKN45, and most of them were located in the cytoplasm (**Figure 6J**). In order to verify the expression of RGS2 in gastric cancer, we found that RGS2 was expressed to varying degrees in different clinical stages of gastric cancer by immunohistochemical staining, and with the increase of staging, the more RGS2 deposition (**Figure 6K**).

### 3.8 Biological significance of RGS2 in other cancer types

**Figure 7:**
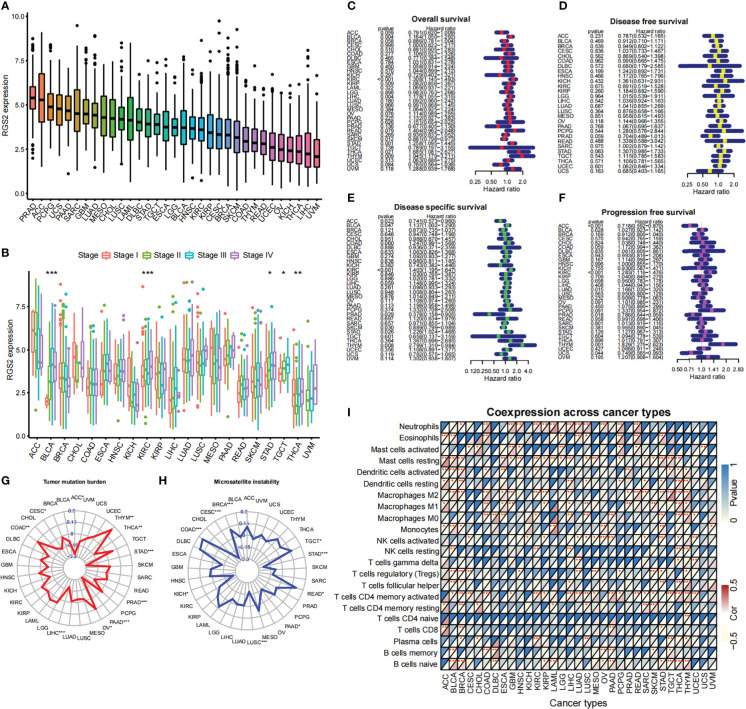
Biological significance of RGS2 in pan-cancer **(A)** RGS2 expression level in multiple tumors **(B)** Correlation analysis between RGS2 expression level and tumor stage **(C)** Relationship between RGS2 expression level and OS **(D)** Relationship between RGS2 expression level and disease-free survival **(E)** Relationship between RGS2 expression level and Disease specific survival **(F)** Relationship between RGS2 expression level and Progression free survival **(G)** Correlation analysis between RGS2 and Tumor mutation burden (TMB) **(H)** Analysis of the correlation between RGS2 and Microsatellite instability (MSI) **(I)** The relationship between RGS2 and immune cell infiltration. *p<=0.05, **p<=0.01,***p<=0.001.

A pan-cancer analysis has shown that *RGS2* is expressed in many tumor types (**Figures 7A, B**). We found that the expression of *RGS2* in the overall survival time (OS) was significantly correlated with the survival rates of BLAC, KIRC, LIHC, SKCM, STAD, THCA, and THYM (**Figure 7C**). There was also no significant difference in the expression of *RGS2* between cancer and disease-free survival (DFS) groups (**Figure 7D**). There was a correlation between disease-specific survival and ACC, BLCA, KIRC, PRAD, SKCM, STAD, and THYM (**Figure 7E**), and also a significant correlation between progression-free survival and ACC, KIRC, and THYM (**Figure 7F**). We analyzed the correlation between RGS2, TMB, and MSI, and found that it was significantly correlated with TGCT, STAD, PAAD, COAD, and CESC, suggesting that it can be used as a basis of detection for immunotherapy of the above tumors (**Figures 7G, H**). Finally, we found that *RGS2* was closely related to the level of immune cell infiltration in most tumors, suggesting that *RGS2* participates in the regulation of the tumor immune response in the tumor microenvironment (**Figure 7I**).

## 4 Discussion

Rapid proliferation and insufficient angiogenesis of tumor cells lead to hypoxia, low pH levels, and depletion of nutrients in the tumor microenvironment (15, 16). Therefore, tumor cells show unique metabolic characteristics that are distinct from those of normal cells. Tumor cells deal with a variety of adverse microenvironments by reprogramming their metabolism, and blocking carcinogenic signals to maintain their proliferating state and survival. Abnormal energy metabolism is thus a hallmark of cancer, which indicates that the metabolism of carbohydrates, lipids, and amino acids in tumor cells is significantly different from that in normal cells. Fatty acid metabolism was previous shown to maintain tumorigenesis, disease progression, and therapeutic resistance by enhancing lipid synthesis, storage, and decomposition (17, 18). Recently, increasing attention has been paid to the role of membrane fatty acids (with respect to e.g. the ratio of saturated fatty acids, monounsaturated fatty acids, and polyunsaturated fatty acids) in promoting cell survival, limiting lipotoxicity, and iron-dependent cell death (19–21). Here, eight fatty acid metabolism-related genes related to gastric cancer prognosis were identified based on an analysis of genomic information of 373 STAD samples and 32 paracancerous tissue samples using univariate COX regression, clustering, and principal component analyses. A model called “FRAS” was constructed, and the score calculated using this model (FRAS score) was found to be closely related to increased immune cell infiltration, genomic instability, immune escape and sensitivity of immune checkpoint inhibitor (ICIs). This fatty acid metabolism-related model was comprehensively evaluated as well. The fatty acid prognostic risk score model was found to be able to independently predict the prognosis of patients with gastric cancer, and effectively distinguish the sensitivity of patients to chemotherapeutic drugs. In addition, the relationship between the prognostic risk score model and characteristics of TME cell infiltration was studied. The prognostic risk score model was found to identify patients with gastric cancer who are suitable for anti-CTLA4 antibody immunotherapy sucessfully, and thereby also indicated that fatty acid metabolism is crucial for shaping individual TME characteristics. These findings may provide a new perspective for exploring the mechanisms of fatty acid metabolism and treatment of gastric cancer.

Rapidly proliferating tumor cells show a high affinity for lipids and cholesterol by increasing exogenous lipid uptake, or by overactivating their biosynthetic pathways (22). Therefore, fatty acid synthesis (FAS) inhibitors, especially fatty acid synthase (FASN), have been the focus of cancer treatment studies (23–25). *RGS2, DUSP1, CXCR4, FNDC1, SNCG, SLCO2A1, APOD*, and *GPX38* were selected to construct this risk model. This model can predict the prognosis of patients with gastric cancer more accurately that a single clinical variable, which may be helpful for clinicians in making clinical decisions. The model was used to classify patients with stage G2/G3, patients aged > 65 years and< 65 years, and patients with *Helicobacter pylori* infection into two groups. This was found to have a significant impact on prognosis, as it confers the advantage of using genetic characteristics in predicting clinical grouping and prognosis.

Gastric cancer patients also develop drug resistance eventually, even though 60% of them are sensitive to chemotherapy. This leads to a 5-year survival rate of less than 10% (26–28). Therefore, understanding the mechanism of chemotherapy resistance in gastric cancer cells is important for improving the prognosis and survival rate. Previous studies have revealed that some cancer cells require fatty acid oxidation to provide energy that is required to maintain the stem cell state. Studies on resistance of breast tumor stem cells (BCSCs) to chemotherapy have found that JAK/STAT3 signaling systems help breast cancer cells maintain their stem cell status, and resistance to chemotherapy by promoting fatty acid oxidation (29, 30). Animal experiments have further confirmed that drugs that inhibit the JAK/STAT3 signaling system can greatly reduce the population of stem cells in breast cancer, and improve the efficiency of chemotherapy (31, 32). Here, we further analyzed the relationship between the develop fatty acid metabolism-related risk score and chemotherapy resistance in gastric cancer cells, and identified significant differences in sensitivity to chemotherapeutic drugs between the high- and low-score groups. Specifically, bortezomib, elesclomol, and nilotinib showed better therapeutic effects in the low-score groups. Targeting of the fatty acid metabolism may thus be a new strategy for reversing drug resistance in gastric cancer cells.

The G protein signal transduction regulatory factor (RGS) gene family, which includes negative regulators of G protein-coupled receptors, are potential drug targets for the treatment of malignant tumors (33, 34). RGS is a large family of genes with multiple functions (35–37). These proteins share an RGS domain with a conserved core that includes 130 amino acid residues, which can directly bind to the activated G-α subunit to inactivate GTP, and thus help negatively regulate GPCR-related signaling pathways (38–40). RGS gene has been proved to be closely related to the occurrence and development of many systemic diseases and cancers (41–43). Here, we analyzed the role of RGS2, in the tumor microenvironment in gastric cancer, and also in other cancer types for the first time. The results showed that the expression of *RGS2* was correlated with interstitial and immune scores. Therefore, we speculate that RGS2 participates in the occurrence and development of gastric cancer by affecting the migration of immune cells. Moreover, we also found that the TMB score of the RGS2 high-expression group was lower than that of the low-expression group, and the TIDE score was higher than that of the low-expression group. This indicates that it is more difficult for gastric cancer patients to benefit from immunotherapy, and have a worse prognosis. High expression levels of *RGS2* were detected by Western blot analysis, which indicates a role of RGS2 in the progression of gastric cancer.In gastric cancer, the deposition of RGS2 increased with the increase of clinical stage. Therefore, in the microenvironment of gastric cancer, RGS2 may predict a poor prognosis. *RGS2* expression in various tumor types was also found to be significantly correlated with survival, clinical stage, immune score, TMB score, and MSI. Therefore, RGS2 could be used as a new tumor marker as well.

However, our study has suffered from some limitations as well. For example, further research is still needed to reveal how fatty acid-related genes affect immune cell infiltration and genomic instability in gastric cancer. In addition, as this study mainly used online datasets for analysis, more clinical data supplement is necessary.

## 5 Conclusion

In conclusion, we analyzed here the expression of fatty acid metabolism-related genes in gastric cancer, and constructed a model based on fatty acidification to calculate a disease risk score for gastric cancer. Our analysis revealed that FARS score in gastric cancer is closely related to tumor mutation load, genomic instability, ICIs treatment response, immune cell infiltration, and immune escape. This score provides with a new tool for the diagnosis and treatment of gastric cancer, and the genes related to FARS may become new tumor markers or therapeutic targets. In general, the FARS score developed in this study can be used as a potential molecular classification tool for gastric cancer, and thus help identify immune infiltration and genomic instability patterns in gastric cancer. FARS can also be used to evaluate response of patients to ICIs treatment.

## Data availability statement

The original contributions presented in the study are included in the article/**Supplementary Material**, further inquiries can be directed to the corresponding author.

## Ethics statement

The study design was approved by the Internal Audit and Ethics Committee of the Second Affiliated Hospital of Harbin Medical University (No: KY2021-075). The patients/participants provided their written informed consent to participate in this study.

## Author contributions

We would like to thank all the participants and staff for their valuable contributions. SY, BS and WL contributed equally as co-first authors. XZ conceived and designed the study; HY and NL drafted the manuscript. All authors contributed to the article and approved the submitted version.

## Supplementary material

Supplementary Table 1 The regression coefficient of each gene

Supplementary Figure 1 Differential expression of fatty acid related genes in different clinical characteristics **(A)** Difference of fatty acid related gene expression among Age groups of gastric cancer **(B)** Difference of fatty acid related gene expression among gastric cancer Grade groups **(C)** Difference of fatty acid related gene expression among gastric cancer Gender groups **(D)** Difference of fatty acid related gene expression among gastric cancer Stage groups

Supplementary Figure 2 Distribution of different groups among subtypes in clinical characteristics. **(A)** Correlation analysis between fatty acid subtypes and different clinical features. **(B)** KM curve analysis of 8 genes (8 genes screened after lasso-cox)
