# Supplementary figures and images for: Fatty acid metabolism is related to the immune microenvironment changes of gastric cancer and RGS2 is a new tumor biomarker
Source: Front Immunol. 2022 Dec 14;13:1065927. doi: 10.3389/fimmu.2022.1065927 (PMC9797045; doi:10.3389/fimmu.2022.1065927)

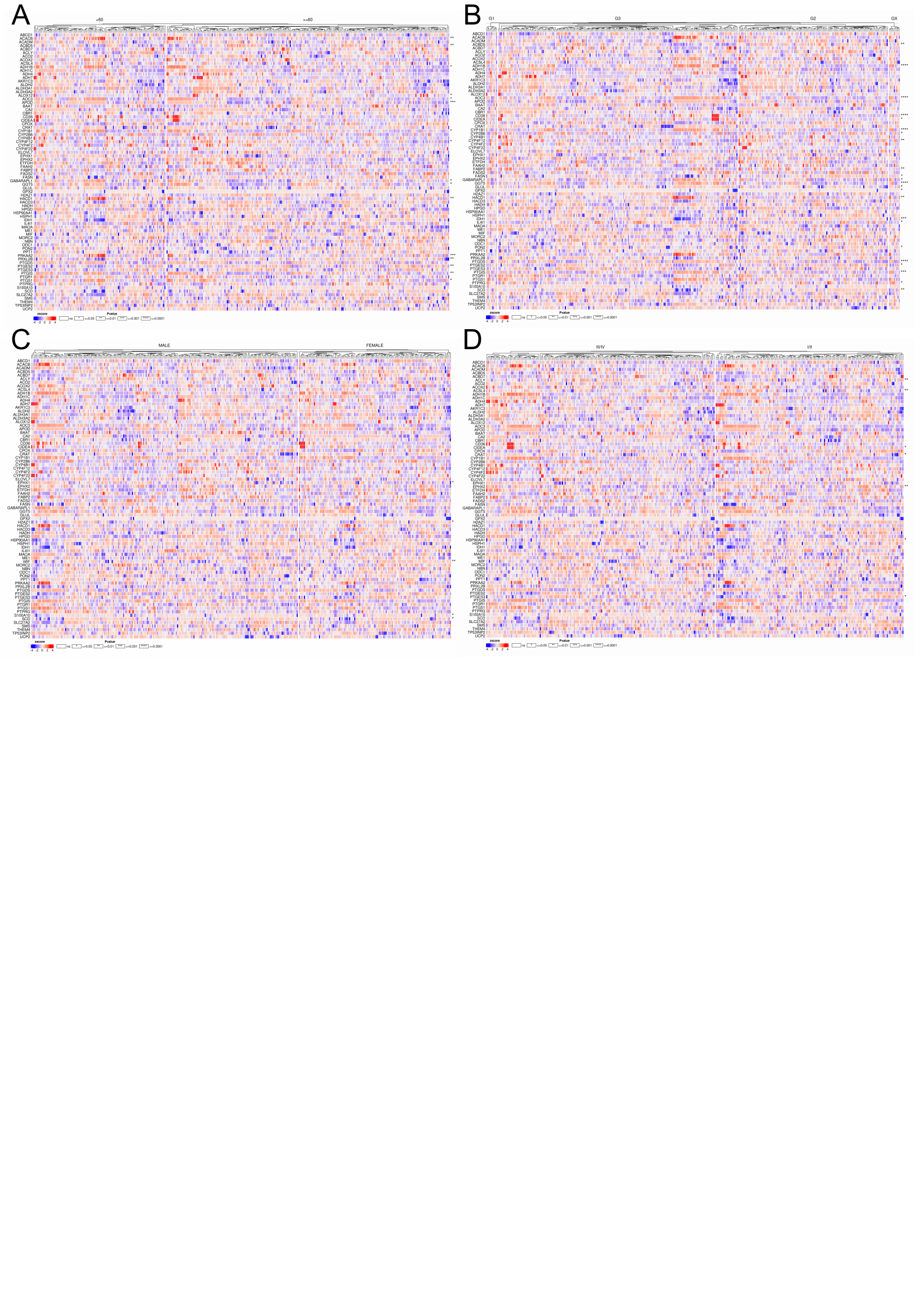

Supplement: Supplementary Figure 1 — Differential expression of fatty acid related genes in different clinical characteristics (A) Difference of fatty acid related gene expression among Age groups of gastric cancer (B) Difference of fatty acid related gene expression among gastric cancer Grade groups (C) Difference of fatty acid related gene expression among gastric cancer Gender groups (D) Difference of fatty acid related gene expression among gastric cancer Stage groups [file Image_1.tif]

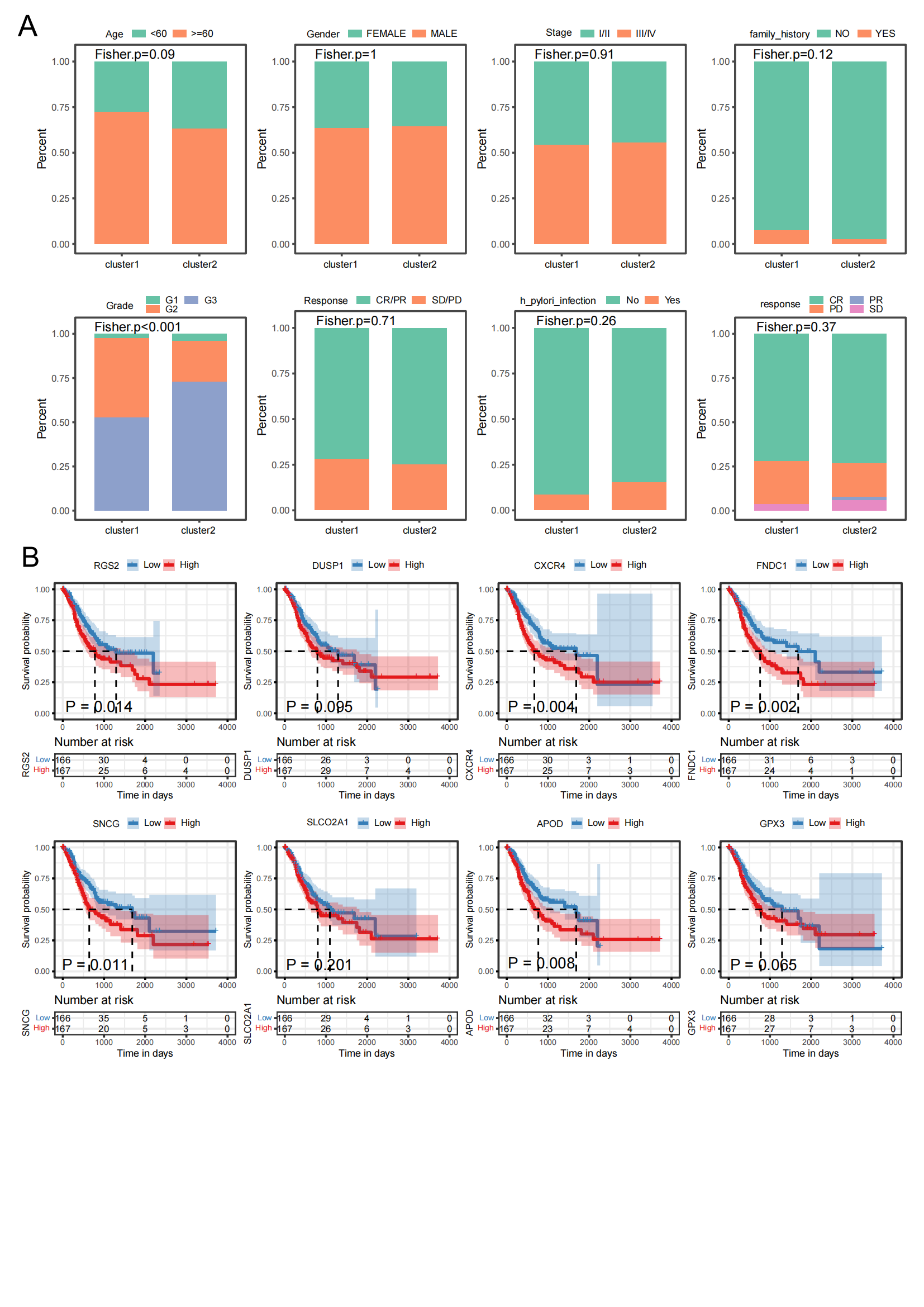

Supplement: Supplementary Figure 2 — Distribution of different groups among subtypes in clinical characteristics. (A) Correlation analysis between fatty acid subtypes and different clinical features. (B) KM curve analysis of 8 genes (8 genes screened after lasso-cox) [file Image_2.tif]
